# Experimental and Numerical Analysis of Sandwich Structures

**DOI:** 10.3390/ma19020386

**Published:** 2026-01-18

**Authors:** Zbigniew Pozorski, Jörg Lange, Agnieszka Sabik

**Affiliations:** 1 Institute of Structural Analysis, Faculty of Civil and Transport Engineering, Poznan University of Technology, ul. Piotrowo 5, 61-131 Poznań, Poland; 2 Institute for Steel Construction and Materials Mechanics, Technical University of Darmstadt, 64287 Darmstadt, Germany; j_lange@stahlbau.tu-darmstadt.de; 3 Department of Mechanics of Materials and Structures, Faculty of Civil and Environmental Engineering, Gdańsk University of Technology, ul. Gabriela Narutowicza 11/12, 80-233 Gdańsk, Poland; agnieszka.sabik@pg.edu.pl

Sandwich structures have become a cornerstone of modern engineering, playing a pivotal role in aerospace, automotive, marine, and civil applications [1,2,3,4]. Their unique combination of light weight, high stiffness, and excellent energy absorption stems from their characteristic design: two strong face sheets bonded to a lightweight core. The ever-increasing performance requirements of advanced engineering industries demand continual innovation in both the experimental characterization and numerical modeling of these intricate systems. This Special Issue (SI), titled “Experimental and Numerical Analysis of Sandwich Structures”, presents current problems concerning the mechanics of sandwich structures, examples of new applications and modern research methods, and possible new areas of implementation. It explores recent advances in the experimental and numerical analysis of sandwich structures, highlighting key research achievements and charting a course for future work. This Editorial summarizes the nine research articles included in the SI.

Experimental investigations are vital for elucidating the complex failure mechanisms and mechanical behavior of sandwich structures under various loading regimes [5,6,7]. In recent years, a multitude of studies have focused on analyzing the bending, shear, and impact responses of novel sandwich systems, often incorporating innovative core or face sheet materials [8,9,10]. For instance, tests on sandwich beams subjected to bending have shed light on the intricate interplay between face sheet and core properties, often revealing modes of failure such as face wrinkling, core shear, or debonding at the interfaces. Much important information on the mechanisms of destruction is also provided by above-standard material tests [11]. It was demonstrated in [12,13,14] that by manipulating the core configuration, it is possible to significantly alter the mechanical performance, especially regarding energy absorption and failure patterns. Furthermore, nonstandard core materials, including 3D-printed lattice structures and advanced foams, have been investigated, revealing that their tailored architecture can dramatically enhance both strength and ductility [15,16,17].

Besides traditional static loading experiments, dynamic and fatigue testing have gained prominence [18]. Advanced testing protocols, often involving drop weight impact or cyclic loading, are crucial for simulating real-world operating environments. The low-velocity impact performance of sandwich panels with novel composite face sheets was investigated in [19], finding substantial improvements in energy absorption compared to conventional systems. Moreover, full-field measurement techniques such as digital image correlation (DIC) and acoustic emission monitoring are revolutionizing the way deformation, damage initiation, and propagation are detected and analyzed during testing. By capturing high-resolution strain fields and real-time failure events, these methods provide unparalleled insight into local and global phenomena, enabling the fine-tuning of sandwich design at the micro and macro scales [20,21,22].

In parallel with experimental advances, numerical modeling has emerged as an indispensable tool for predicting the behavior of sandwich structures. Finite element analysis (FEA), in particular, has reached new levels of sophistication. Contemporary models account for complex phenomena such as plasticity, progressive damage, delamination, and even the effects of manufacturing defects [23,24]. Researchers increasingly employ multi-scale modeling strategies, bridging the gap between microstructural core design and macro-scale structural response [25,26]. Recent studies have shown that by incorporating accurate material laws, interfacial properties, and detailed geometric features, numerical simulations can replicate experimental results with impressive fidelity [27,28]. Another important issue is the proper determination of material parameters, which is particularly important in the case of analysis of layered structures subjected to extreme thermal actions [29]. The appropriate numerical model allows us to achieve predictive capability, which is crucial for finding design solutions without the need to conduct numerous and expensive physical tests, including fire tests [30].

Nevertheless, the credibility of numerical approaches hinges upon rigorous validation and calibration against experimental measurements. Discrepancies between predicted and observed behavior often arise from simplifications in model assumptions, inaccuracies in input data, or neglected failure modes. For example, the role of face/core debonding or local wrinkling might be underestimated unless explicitly modeled. Collaborative experimental–numerical studies, such as those synthesizing extensive test data with high-fidelity simulations, have proven instrumental in refining models and enhancing their practical applicability [31,32,33,34,35].

The intersection of experimental and numerical methods is fueling a wave of innovation in sandwich structure design. Modern research efforts are increasingly focused on bio-inspired architectures, multifunctional materials, and the integration of sensing or energy-harvesting capabilities [36,37,38,39,40]. Three-dimensional printing and additive manufacturing technologies facilitate unprecedented control over core geometry, leading to complex, lattice-based designs that outperform traditional foams in specific strength, stiffness, and impact resistance [41,42,43]. Moreover, hybrid core configurations, combining materials such as metals, polymers, and ceramics, are being explored to leverage their complementary attributes, for instance, optimizing for both thermal stability and impact tolerance [44,45]. The synergy between material development, advanced modeling, and bespoke testing protocols is therefore vital for pushing the frontiers of sandwich structure applications [46].

Emergent challenges, particularly in the context of sustainability, are prompting the adoption of recyclable or renewable materials in both face sheets and cores [47]. Life-cycle analysis, durability testing, and predictive modeling under varied environmental exposures are key themes in ongoing research, ensuring that new sandwich systems are not only high-performing but also environmentally conscious [48,49,50].

The experimental and numerical analysis of sandwich structures remains a dynamic and rapidly evolving field. Integration of cutting-edge testing methodologies with increasingly detailed simulation tools is unraveling the nuances of these complex materials, enabling the design of lighter, stronger, and more adaptive systems. As industries continue to demand higher efficiencies and multifaceted performance, interdisciplinary collaboration will be indispensable, merging advances in manufacturing, materials science, and computational mechanics. The ongoing synergy between experiments and simulations promises to unlock new realms of possibility, ensuring sandwich structures remain at the forefront of advanced engineering solutions for decades to come.
